# A new chapter of healthy indoor air: antiviral air treatments

**DOI:** 10.15252/emmm.202318710

**Published:** 2023-11-16

**Authors:** Irina Glas, Shannon C David

**Affiliations:** ^1^ Institute of Medical Virology University of Zurich Zürich Switzerland; ^2^ Environmental Chemistry Laboratory, School of Architecture, Civil and Environmental Engineering Ecole Polytechnique Fédérale de Lausanne (EPFL) Lausanne Switzerland

**Keywords:** Microbiology, Virology & Host Pathogen Interaction

## Abstract

Aerosol transmission remains a major challenge for the control of respiratory viruses. To date, prevention strategies include masks, vaccinations, physical distancing, travel restrictions, and lockdowns. Such measures are effective but come with heavy societal burdens and rely on public compliance. Additionally, most are simply not suitable as long‐term measures. Other strategies evolve around the concept of improved indoor air quality and involve ventilation, relative humidity (RH) control, and air filtration. Unfortunately, natural ventilation increases exposure to airborne pollutants and vector‐borne diseases, and incurs substantial energy losses in colder months. Mechanical ventilation concepts, including regular air changes and filtration, are effective but costly, and often require expensive engineering solutions and widespread renovations. Alternative options to reduce the spread of emerging and seasonal infections are sorely needed. In this issue of EMBO Molecular Medicine, Styles *et al* (2023) describe the use of propylene glycol (PG) to inactivate infectious bioaerosols and virus‐containing droplets deposited on surfaces.

## Graphical abstract

SC David and I Glas discuss potential use of propylene glycol to inactivate infectious bioaerosols and virus‐containing droplets in indoor spaces as reported by R Edgar and colleagues, in this issue of EMBO Mol Med.

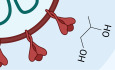

There are many studies investigating different strategies for control of airborne bioburdens in indoor spaces (Fig 1). PG, as one of them, is already approved for widespread applications by the US Food and Drug Administration (FDA) and European Medicines Agency (EMA), and is used within the cosmetic, food, and pharmaceutical industries as vehicle and humectant (including oral, topical, intravenous, and nebulized drug delivery). This work shows PG has virucidal effects against a broad range of human viruses including influenza A virus (IAV), SARS‐CoV‐2, Epstein–Barr virus, multiple pseudoviruses, and also the non‐enveloped rotavirus, which indicates this compound could be additionally applicable to mitigating enteric pathogen outbreaks. Furthermore, PG vapor was highly effective at inactivating aerosolized IAV and SARS‐CoV‐2 as well as IAV deposited upon plastic, steel, glass, and aluminum surfaces, highlighting its dual potential to simultaneously reduce viral burdens in air and on surfaces. Mice infected with PG‐ treated IAV showed enhanced survival and reduced clinical scores compared with control IAV‐infected animals, while PG alone was well‐tolerated and showed no adverse symptoms in animals, consistent with the long‐established safety of this compound.

**Figure 1:**
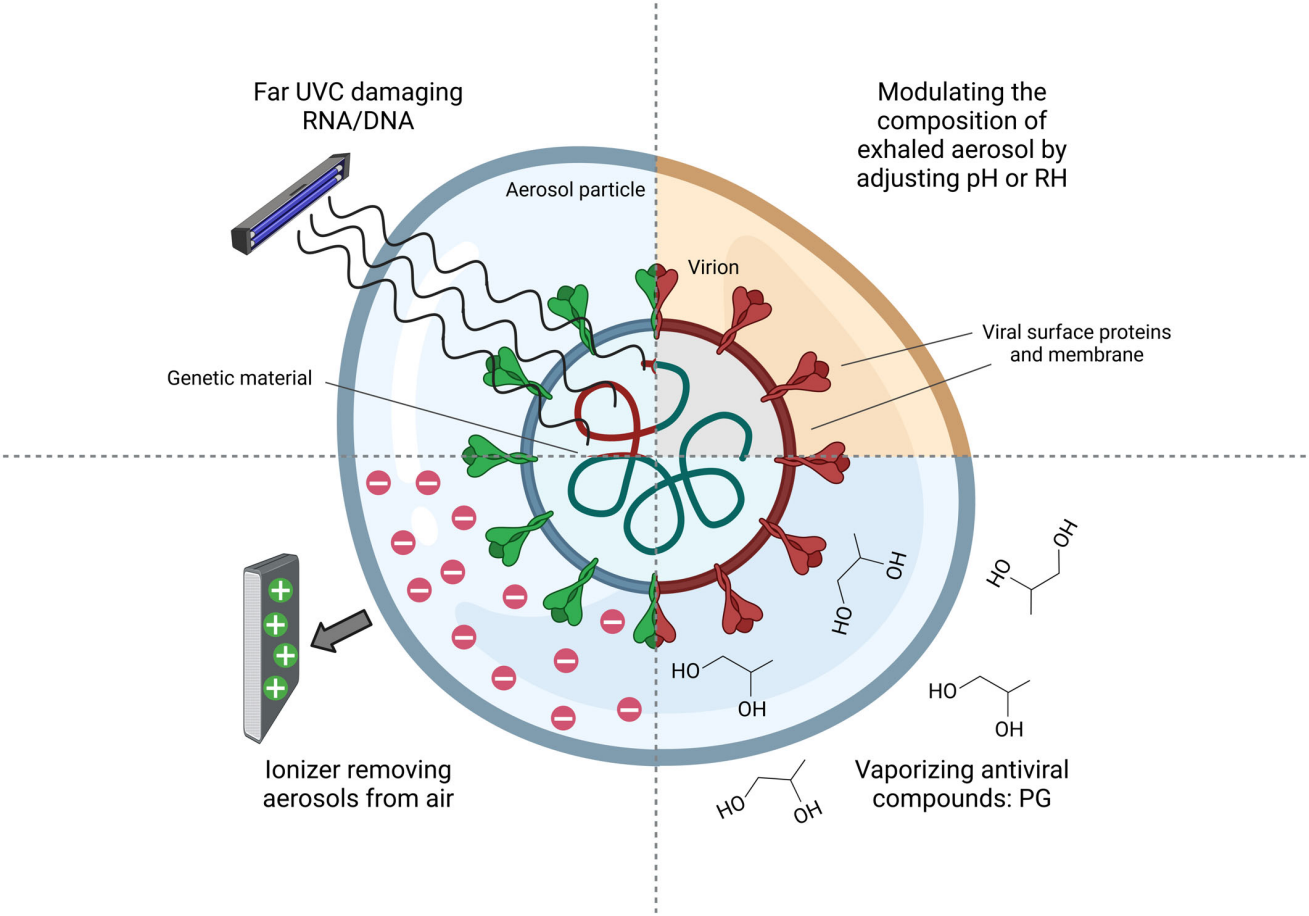
Antiviral air treatments under current research Depicted is an aerosol particle containing an exemplary enveloped virion, consisting of a membrane, surface proteins, and genetic material. Antiviral air treatments can damage the genetic material of aerosolized viruses (far UVC light), induce a damaging aerosol microenvironment (RH and pH modulation), or affect the viral membrane and surface proteins (PG). Damaged viral components are shaded red. Alternatively, air ionization physically removes aerosols from air via electrostatic interactions (created with biorender.com).

Overall, authors present a compelling and rigorous study that showcases the potential of this antiviral for broad‐spectrum pathogen management in indoor spaces. Although the exact inactivation mechanisms of PG remain to be elucidated, work shown here already suggests that PG disrupts viral membranes and structural proteins. These findings, together with the broad range of affected viruses, indicate a low likelihood of resistance development. Authors propose that PG in nasal sprays or nebulizers could be used to protect vulnerable individuals, while direct inactivation of airborne viruses by PG vapor could reduce the overall infectious burden and transmission rates in clinical and commercial settings.

Other studies have investigated physical options for airborne virus inactivation. UVC, for example, is a well‐known and widely used disinfectant. Its RNA and DNA damaging properties affect bacteria, viruses, as well as eukaryotic cells. Specifically, far UVC light is not able to penetrate the skin or eyes and is considered safe. Buonanno *et al* (2020) found that far UVC light (222 nm) efficiently inactivates aerosolized coronaviruses, estimating a 90% reduction of infectivity within 8 min. Installing far UVC lamps could reduce overall viral loads in room air and limit transmission. As an alternative physical treatment option, Hagbom *et al* (2015) utilized a portable ionizer to render airborne virus‐containing particles negatively charged, resulting in electrostatic attraction to a positively charged collector plate. This strategy was effective at physically pulling airborne calicivirus, rotavirus, and IAV from an air chamber 19 m^3^ in size, and was also shown to prevent airborne transmission of IAV between live animals. This strategy has the added benefit of pandemic/epidemic monitoring, as captured viruses were easily removed and identified by sequencing. This would provide an additional surveillance method for anticipation of viral outbreaks, for example, in hospital settings.

However, ionizers may be inefficient at removing larger droplets or disinfecting surfaces, and physical shading protects infectious aerosol from UV light. This leaves room for alternative solutions, like RH control, though the influence of RH on aerosolized virus stability is controversial. Kormuth *et al* (2018) found RH plays a minor role in IAV infectivity when respiratory fluids are contained in the aerosol matrix, as opposed to a strong RH effect for aerosols composed of cell culture medium (more commonly used in aerosol experiments). Conducting *in vivo* experiments in a guinea pig model, Lowen *et al* (2007) showed that aerosol transmission of IAV is dependent on RH. Whether RH influences virus viability in aerosols or affects host defense mechanisms is not well understood. A more recent *in vivo* study by Ganti *et al* (2022) researching aerosol transmission of SARS‐CoV‐2 found only a minor relation between RH and transmission, suggesting differences in RH susceptibility of different viruses. Nevertheless, lowest transmission rates and airborne virus stability are usually measured at intermediate RH (40–60%), emphasizing its important role in healthy indoor air. Recent studies also indicate that mild pH adjustment of room air could be a rapid way to reduce infectious bioburdens within indoor spaces. Depending on the virus's pH sensitivity, a slight pH reduction (suitable for IAV) shown by Luo *et al* (2023) or pH increase (suitable for SARS‐CoV‐2) shown by Oswin *et al* (2022) could dramatically reduce stability of aerosol‐borne viruses. Depending on the most prominent virus, air pH could be modulated during peak infection periods. Alternatively, these findings could be applied to development of nasal sprays for more targeted protection. Low‐pH nasal sprays were effective at reducing IAV infection in animals (Rennie *et al* (2007)), well‐tolerated in humans, and reduced viral shedding of the similarly acid‐sensitive rhinovirus after experimental human infection (Gern *et al* (2007)). Much like PG, pH‐specific sprays could be utilized by at‐risk populations (e.g., elderly, immunocompromised patients, etc.), or could be self‐administered by infected individuals to reduce community spread and protect healthcare workers.

While healthy air has been studied for decades, adding antiviral air treatments to the concept is a comparably new development. Viruses are vulnerable as they transit between hosts, and reducing infectivity before an expelled particle reaches a new host would be an immense step forward in infection management. Strategies discussed here target a broad range of viruses and intervene independently of individual preventive choices like masks and vaccinations. They would be particularly beneficial during peak seasons or in high‐risk areas (e.g., schools, hospitals, and aged‐care facilities), although it is hoped that clean air strategies will be incorporated into all standard building codes and practices in the future. These antiviral methods would also be effective regardless of an individual realizing they are infected, increasing viral control prior to presentation of any tangible symptoms. Of course, a high level of human safety for long‐term exposure must be established prior to any widespread adoption of a new air treatment, although the wide scope of treatments currently being investigated is promising. Thus far, intermediate RH (40–60%) is the only option known to be safe and additionally healthy for the human respiratory tract. Treatments deemed unsuitable for human spaces could instead be applied to animal settings. Animals in mass production are typically not held for extended time periods, rendering long‐term exposure less of an issue. Furthermore, implementation of these measures in settings with high zoonotic risks, for example, animal markets, would considerably reduce virus spillover into the human population.

## Disclosure Statement & Competing Interests

The authors state they have no competing interests or disclosures

## Author contributions

**Irina Glas:** Visualization; writing – original draft; writing – review and editing. **Shannon C David:** Conceptualization; writing – original draft; writing – review and editing.
